# Application of Whole Exome Sequencing in Six Families with an Initial Diagnosis of Autosomal Dominant Retinitis Pigmentosa: Lessons Learned

**DOI:** 10.1371/journal.pone.0133624

**Published:** 2015-07-21

**Authors:** Berta Almoguera, Jiankang Li, Patricia Fernandez-San Jose, Yichuan Liu, Michael March, Renata Pellegrino, Ryan Golhar, Marta Corton, Fiona Blanco-Kelly, Maria Isabel López-Molina, Blanca García-Sandoval, Yiran Guo, Lifeng Tian, Xuanzhu Liu, Liping Guan, Jianguo Zhang, Brendan Keating, Xun Xu, Hakon Hakonarson, Carmen Ayuso

**Affiliations:** 1 Center for Applied Genomics, The Children's Hospital of Philadelphia, Philadelphia, PA, 19104, United States of America; 2 BGI-Shenzhen, Shenzhen 518083, China; 3 Department of Genetics and Genomics, IIS-Fundacion Jimenez Diaz, 28040, Madrid, Spain; 4 Center for Biomedical Network Research on Rare Diseases (CIBERER), ISCIII, Madrid, Spain; 5 Department of Ophthalmology, Fundacion Jimenez Diaz, 28040, Madrid, Spain; Hadassah-Hebrew University Medical Center, ISRAEL

## Abstract

This study aimed to identify the genetics underlying dominant forms of inherited retinal dystrophies using whole exome sequencing (WES) in six families extensively screened for known mutations or genes. Thirty-eight individuals were subjected to WES. Causative variants were searched among single nucleotide variants (SNVs) and insertion/deletion variants (*indels*) and whenever no potential candidate emerged, copy number variant (CNV) analysis was performed. Variants or regions harboring a candidate variant were prioritized and segregation of the variant with the disease was further assessed using Sanger sequencing in case of SNVs and *indels*, and quantitative PCR (qPCR) for CNVs. SNV and *indel* analysis led to the identification of a previously reported mutation in *PRPH2*. Two additional mutations linked to different forms of retinal dystrophies were identified in two families: a known frameshift deletion in *RPGR*, a gene responsible for X-linked retinitis pigmentosa and p.Ser163Arg in *C1QTNF5* associated with Late-Onset Retinal Degeneration. A novel heterozygous deletion spanning the entire region of *PRPF31 *was also identified in the affected members of a fourth family, which was confirmed with qPCR. This study allowed the identification of the genetic cause of the retinal dystrophy and the establishment of a correct diagnosis in four families, including a large heterozygous deletion in *PRPF31*, typically considered one of the pitfalls of this method. Since all findings in this study are restricted to known genes, we propose that targeted sequencing using gene-panel is an optimal first approach for the genetic screening and that once known genetic causes are ruled out, WES might be used to uncover new genes involved in inherited retinal dystrophies.

## Introduction

Despite the advances over the last decades on the genetics of inherited retinal dystrophies, molecular diagnosis of this heterogeneous group of diseases is still challenging. The inherited retinal dystrophies include a wide spectrum of diseases caused by more than 190 genes identified so far (RetNet; https://sph.uth.edu/retnet/), and represent the most frequent cause of genetic blindness in the Western world. With an overall prevalence of up to 1 in 4000 individuals worldwide [1], retinitis pigmentosa (RP) is the most common form of inherited retinal dystrophy and accounts for almost half of the patients [2].

RP is a set of inherited progressive and degenerative retinal diseases that lead to loss of vision (reviewed in [2] and [3]). All types of Mendelian inheritance patterns have been described for RP, with autosomal dominant RP (adRP) accounting for 15–25%, autosomal recessive RP (arRP) for 35–50%, and X-linked RP (xlRP) for up to 15% of the families [4]. One particular characteristic of RP is its extreme genetic and allelic heterogeneity, what makes the diagnosis of patients a complex task. As of the last update of the Retinal Information Network (RetNet; https://sph.uth.edu/retnet/), more than 2,800 mutations in 81 genes had been identified to cause RP: 26 in adRP, 52 in arRP and three causing xlRP. To make it even more complex, large phenotypic heterogeneity is also observed, with mutations in the same genes causing different diseases and the same mutation displaying extensive variation in clinical expression, if not clinically distinct entities, among individuals [3]. Symptoms and phenotypes are variable between families and also in different members of the same family, and several genes display incomplete penetrance [2].

Until the advent of Next Generation Sequencing (NGS), molecular diagnosis of RP was mainly based on a combination of arrayed primer extension (APEX) technology of previously known mutations, and Sanger sequencing, which results unaffordable for the screening of all potentially causative genes. Due to its high-throughput nature, NGS is revolutionizing the way disease-causing mutations of Mendelian disorders are identified [5], [6] being able to simultaneously scan multiple genes in a cost-efficiently manner and has proven very productive in RP, with high mutation detection and diagnostic rates [7–11], as well as the discovery of new genes [12–14]. Specifically, targeted capture of known and candidate genes is emerging as the most optimal diagnostic tool for RP [10, 11, 15, 16] with a mutation detection rate of 20–70%, depending on the inheritance pattern and selection criteria [3], [7, 8]. Notably, approximately 50% of the cases of adRP are estimated to harbor mutations in novel genes [3] that might not be captured by gene-panels, and in these cases whole exome sequencing (WES), which targets the complete coding part of the genome, could help identify the missing causative genes. In this study, we used WES in six families with a suspected diagnosis of adRP as a part of a larger effort on the identification of the genetic causes of Mendelian disorders. The application of WES led to the characterization of four of the six families, allowing the reappraisal of the diagnosis in three, and the identification of a novel large deletion in *PRPF31* responsible for the phenotype in a fourth family.

## Materials and Methods

### Subjects

A total of 66 individuals from six large unrelated Spanish families from the Fundacion Jimenez Diaz University Hospital, with an initial diagnosis of non-syndromic adRP were included in the current study (S1 Fig) The criteria for the assignment of autosomal dominant inheritance were based on that previously described by Ayuso *et al*. [17].

Thirty-eight members out of the 66 were selected for WES (S1 Fig). Written informed consent was obtained from all individuals involved in the study, and the research was performed in accordance with the tenets of the Declaration of Helsinki and further reviews. Protocols were approved by the Bioethics Committee of the IIS-Fundacion Jimenez Diaz.

Genomic DNA was extracted from peripheral blood lymphocytes and/or saliva (Oragene containers, DNA Genotek) using standard methods. Index cases enrolled were previously screened for known causes of adRP using a combined strategy of molecular tools: Single Strand Conformation Polymorphism (SSCP), CG-clamped Denaturing Gradient Gel Electrophoresis (DGGE), genotyping ADRP Chip (Asper Biotech, Tartu, Estonia), Sanger sequencing of prevalent adRP genes [18], [19], [20] and a NGS-based approach with a custom panel for 73 genes related to retinal dystrophies [10, 20]. (See S1 and S2 Tables).

### Clinical evaluation

The clinical ophthalmic evaluation included the assessment of visual acuity (VA), intraocular pressure, ocular motility, pupillary reaction, biomicroscopic slit-lamp examination, and dilated fundus examination in all members of the six families. Visual function was performed by static perimetry, D15 panel testing, and Ganzfeld electroretinography according to the International Society for Clinical Electrophysiology of Vision (ISCEV) Standards [21] with a UTAS 2000 system (LKC Technologies, Gaithersburg, USA) and jet electrodes.

RP diagnosis was made in patients with night blindness, progressive visual field constriction, poor VA in advanced stages, and confirmed by altered or abolished electroretinogram (ERG) responses [22].

### Whole exome sequencing

DNA samples were subjected to library construction using Agilent Sure Select Human All Exon kit version 2 covering 46MB of coding region (Agilent Technologies, Santa Clara, CA, USA), and sequenced on HiSeq 2000 instruments (Illumina, San Diego, CA, USA). Default parameters predefined in the Illumina sequencing workflow were applied to call bases from raw images, which produced raw sequencing reads that were mapped against the human reference genome (UCSC hg19), using the Burrows–Wheeler alignment tool [23]. Genome Analysis Tool Kit version 1.4 [24] was integrated with own scripts to design a variant calling pipeline for genomic variant detection, including single nucleotide variants (SNVs) and small insertions/deletions (*indels*). ANNOVAR [25] was used for variant functional annotation.

### Variant prioritization

Causative variants were first searched among all SNVs and *indels* and whenever no potential candidate emerged, copy number variant (CNV) analysis was performed using WES data. Variants or regions harboring a candidate variant in genes previously associated with retinal dystrophies or with expression in the retina were prioritized and segregation of the variant with the disease was further assessed. Databases used for such prioritization were the Retinal Information Network Database (RetNet; https://sph.uth.edu/retnet/) and the Human Gene Mutation Database (HGMD; www.hgmd.org/).

SNVs and *indels* in coding regions and potentially functional (nonsynonymous, splice acceptor and donor site SNVs, or frameshift *indels*) were considered for the analysis. From those, only novel variants or those with a MAF<1% in a cohort of more than 8,000 control individuals (1,000 Genomes Project, April 2012 release; 6,503 exomes from NHLBI GO Exome Sequencing Project-ESP6500SI; http://evs.gs.washington.edu/EVS/), and 669 in-house whole-exomes) were kept for the subsequent analyses.

CNV analysis was performed using the standard Exome Hidden Markov Model (XHMM) [26]. Briefly, target regions with extreme GC content (<10% or >90%), and low complexity regions were filtered out. Then, read depths of all targets and samples were calculated with GATK [24] and normalized using principal component analysis (PCA) to remove inherent biases in sample preparation and sequencing. Samples with extreme variability in normalized read depth were removed. Finally, per-sample CNV detection with a Hidden Markov Model was performed and quality metrics assigned to all samples for detected CNVs.

### Genetic characterization of *ORF15* and molecular validation of the candidate variants

#### Sanger sequencing

Sanger sequencing was used to validate the candidate SNVs and *indels* selected, their segregation in the families and also to sequence the 3' end of a highly repetitive region of exon open reading frame 15 (*ORF15*) of RPGR in family RP-0502. All primers were designed using Primer3 (*frodo*.*wi*.*mit*.*edu*/). PCR products were enzymatically purified with ExoSAP-it (USB, Affymetrix), sequenced on both strands using Big Dye Terminator Cycle Sequencing Kit v3.1 Kit (Applied Biosystems) and resolved on an automated sequencer (ABI 3130xl Genetic Analyzer, Applied Biosystems).

For the mutation screening in *ORF15*, 13 primer sets were used for the amplification of exon 14 (*ORF14*) and exon 15 (*ORF15*) of RGPR (RefSeq NM_001034853) (S3 Table) [27]. PCR amplifications were done in 50-μL reactions using FastStart polymerase (Roche) according to the recommended protocols. PCR conditions were: 95°C for 5 minutes, followed by 35 cycles of pre-incubation at 95°C for 1 minute, annealing for 1 minute at the indicated temperature in S3 Table and extension at 72°C for 1 minute. After amplification, PCR products were enzymatically purified with ExoSAP-it (USB, Affymetrix) and sequenced on reverse strand using Big Dye Terminator Cycle Sequencing Kit v1.1 Kit (Applied Biosystems) in presence of 10% of betaine (Sigma). PCR products were purified on a 96-well multiscreen filter plate (Montage SEQ96 Sequencing Reaction Cleanup Kit, Millipore, Bedford, MA) and resolved on an automated sequencer (ABI 3130xl Genetic Analyzer, Applied Biosystems).

#### CNV validation

Validation of the large deletion in the gene *PRPF31* was performed in the two affected and six unaffected members of family RP-0777 using quantitative PCR (qPCR) with two different methods: TaqMan assays using the predesigned probes Hs01877341_cn (chr19:54,618,875) and Hs01993463_cn (chr19:54,619,056) (Applied Biosystems TaqMan Copy Number Assays, Life Technologies, Inc.) and the Universal Probe Library (UPL; Roche, Indianapolis, IN) with slight modifications of what was previously described in [28] and [29]. Briefly, primer and UPL probe combinations were designed against *PRPF31* genomic DNA sequence using the Probe Finder v2.49 software (Roche, Indianapolis, IN). Five assays spanning the length of the gene were selected for validation (genomic coordinates of each targeted amplicon listed in Table 1).

**Table 1:** UPL Probes used for validation of *PRPF31* heterozygous deletion. The amplicon position is that reported by UCSC genome browser (hg19) *in silico* PCR tool. All primers are listed 5’ to 3’.

*Gene Symbol-*Probe Name	Gene	Amplicon Position	UPL Probe #	Left Primer^2^	Right Primer^2^
*PRPF31*–345	PRP31 pre-mRNA processing factor 31 homolog	chr19:54,619,134–54,619,198 (exon 1)	55	ggtgagcgactaacgctagaa	cgtggtctccatcacactca
*PRPF31*–3068	PRP31 pre-mRNA processing factor 31 homolog	chr19:54,621,857–54,621,932 (intron 3)	14	ctagcagggggctctagaca	gtcagaatccagcactcttcaa
*PRPF31*–8679	PRP31 pre-mRNA processing factor 31 homolog	chr19:54,627,468–54,627,530 (intron 7)	7	gggaaaaacactcacccaca	gtggtcatctctgggtttcc
*PRPF31*–10932	PRP31 pre-mRNA processing factor 31 homolog	chr19:54,629,721–54,629,783 (intron 8)	17	ctgccctcatcccctctt	cccttgggctctagaggtgt
*PRPF31*–15386	PRP31 pre-mRNA processing factor 31 homolog	chr19:54,634,175–54,634,248 (intron 13)	25	cagtggctgtgcctttcc	gcttcctgtgcgttcttttc
*RPPH1*	RPPH1 ribonuclease P RNA component H1	chr14:20,811,245–20,811,337	30	ccggagcttggaacagact	gtagtctgaattgggttatgaggtc
*GAPDH*	Glyceraldehyde 3-phosphate dehydrogenase	chr12:6,645,563–6,645,625	10	gctgcattcgccctctta	gaggctcctccagaatatgtga
*SNCA*	Synuclein, Alpha	chr4:90,743,466–90,743,537	68	gctgagaagaccaaagagcaa	ctgggctactgctgtcacac

Quantitative PCR was performed on an ABI Prism 7900HT Sequence Detection System (Applied Biosystems, Foster City, CA). Data were evaluated using the Sequence Detection Software v2.4 (Applied Biosystems, Foster City, CA) and further analyzed by the ΔΔCT method. The geometric mean of the CT values for the three control sequences (*GAPDH*, *RPPH1*, and *SNCA*) was calculated and used as the reference value for ΔCT calculations. Hemizygous deletions were determined when the relative copy number value for a specific sample normalized to the reference sample was less than 0.75.

## Results

### SNV and *indel* analysis with further confirmation of family segregation by Sanger sequencing allowed the genetic characterization of three families

Seventeen affected and 21 unaffected members from the six families were subjected to WES. A total of 107.7 GB of data on target genomic regions were generated for the 38 samples, with a mean coverage of target region of 63.67 fold (minimum coverage was 34.85 fold). An average of 50,562 SNVs and 8,476 *indels* were called for the 38 exomes, however, for further variant filtering only variants located in the coding regions and splicing boundaries were considered, thus reducing the number to an average of 18,278 SNVs and 708 *indels*.

Variant filtering was initially performed in the index cases from all six families and then segregation with the disease was assessed in the remaining family members analyzed by WES. SNV and *indel* analysis led to the identification of three previously reported mutations associated with retinal dystrophies in families RP-0107, RP-0858, and RP-0911. A description of the phenotypic features of the affected members of these families and figures with electroretinogram and eye fundus are shown in Table 2 and S2 and S3 Figs.

**Table 2:** Clinical features of the four families genetically characterized in the present study. All the ages are expressed in years. adRP = autosomal dominant retinitis pigmentosa, DOB = date of birth, BE = both eyes, ERG = Electroretinogram, HM = High myopia, LE = left eye, LORD = Late Onset Retinal Dystrophy, LP = Light perception, MA = myopic astigmatism, MD = macular degeneration, MM = myopic maculopathy, NA = not available, NB = night blindness, NR = Non recordable, RE = right eye, RP = retinitis pigmentosa; RPE = Retinal pigment epithelium, VA = visual acuity, VEP = visual evoked potentials, VF = visual field, xlRP = X-linked retinitis pigmentosa.

Family	Subject	Revised diagnosis	Age at diagnosis	Age at onset	Age at time of testing	Visual field	Eye fundus	ERG	Visual acuity	Other
(Gene)	(DOB)			NB/VF/VA					RE/LE	
**RP-0107**	III:2 (1915)	adRP	NA	12/40/35	NA	NA	NA	NA	NA	Myopia (18y) and cataract
*(PRPH2)*	III:6 (1924)	adRP	14	12/14/62	63	Absolute scotoma	Typical RP with macular alteration	NA	NA	Cataract (55y)
	IV:3 (1950)	adRP	NA	40/40/40	NA	NA	Salt-and-pepper pigmentation	NA	NA	
	V:3 (1976)	adRP	NA	NA	NA	NA	Salt-and-pepper pigmentation	NA	NA	
	V:4 (1980)	adRP	17	13/16/20	17	Diffuse relative scotoma	Salt-and-pepper pigmentation	NA	0.7/0.8	Dyschromatopsia
**RP-0777**	II:4 (1966)	adRP	NA	NA	32	Peripheral constriction	Typical RP, no macular affectation in BE	Reduced amplitudes typical of bilateral retinopathy	1.0/1.0	
*(PRPF31)*	III:1 (1934)	adRP	32	27/32/NA	79	Tubular field	Normal vessels and papilla, peripapillar atrophy 360°. No pigmentary lessions in BE	Rods: minimum reduced amplitude and increment of latencies; mix: minimum reduced amplitude in a wave and increment latencies in a and b waves; cones: minimum reduced amplitude b wave and minimum increment latencies in waves; flicker: normal in BE	0.7/0.8	
**RP-0858**	III:4 (1947)	xlRP	43	08/08/08	44	Concentric narrowing	Pale papilla, attenuated retinal vessels, peripheral pigment deposits in BE	NR BE	FC/LP	HM and PSC and altered VEP in BE (56y)
*(RPGR)*	III:6 (1943)	xlRP	NA	NA		NA	NA	NA	NA	Glaucoma
	IV:6 (1965)	xlRP	31	3/23/3	31	Severe concentric narrowing in BE	Typical RP	NR BE	FC/0.4	Strabismus, amblyopia RE, and myopia LE
	IV:8 (1966)	xlRP	37	NA	45	Severe concentric narrowing in BE	Typical RP	NA	0.2/0.1	MM (BE)
	IV:9 (1969)	xlRP	35	Childhood	37	Severe concentric narrowing in BE	Pale papilla, attenuated retinal vessels, macular affectation in BE	Diffuse and severe impairment but not abolished	0.15/0.15	HM, MA, MM
	IV:10 (1974)	xlRP	35	NA	NA	NA	NA	NA	0.8/0.8	HM, MM (BE)
	V:2 (1998)	xlRP	14	NA	NA	Severely affected	Typical RP	NA	0.5/0.5	MA
	V:3 (1996)	xlRP	14	NA	14	NA	NA	NA	NA	NA
**RP-0911**	III:7 (1925)	LORD	60	43/43/40	67	NA	Macular atrophy and bone spicules in periphery	NA	0.5/0.2	Cataract (67y)
(*C1QTNF5*)	IV:1 (1946)	LORD	54	54/54/No	66	Central scotome	Normal papilla, RPE macular atrophy, no pigment	Rods and cones: abnormal amplitudes	0.8/0.8	
	IV:6 (1946)	LORD	NA	61/61/61	67	Central scotome nasal superior	RPE macular atrophy and hipopigmentary rounded areas	Rods: NR, mix: very reduced amplitudes cones and flicker: reduced amplitudes BE	0.1/0.4	Cataract (63y)
	IV:10 (1952)	LORD	59	59/No/No	62	Normal	Macular drusen	Normal	0.9/1	
	IV:11 (1956)	LORD	60	60/60/60	60	Central scotome	RPE macular atrophy, bone spicules in periphery	Rods: very reduced amplitude in b wave; mix and cones: reduced amplitude in a and b waves; cones: reduced amplitude in a and b waves, flicker: reduced amplitude in b wave BE	0.5/0.2	Cataract (56y)
	V:1 (NA)	LORD	NA	NA	NA	NA	NA	NA	NA	NA

Family RP-0107 harbored a missense change in *PRPH2* (NM_000322.4:c556G>A; p.Asp186Asn) [30] and the complete segregation in all five affected and one unaffected members from RP-0107 was further confirmed by Sanger sequencing. RP-0858 carried a known *frameshift* deletion in *RPGR* (NM_000328.2: c.485_486delTT; p.Phe162Tyrfs*4) [31], a gene associated with xlRP. This mutation completely segregated in all 25 members from family RP-0858: it was present in eight cases, including six symptomatic and two asymptomatic female carriers and absent in the 17 unaffected members of the family. In RP-0911, a novel nucleotide change in *C1QTNF5* (NM_015645.3: c.489C>A), leading to the missense mutation p.Ser163Arg previously associated with Late-Onset Retinal Degeneration (LORD, #605670) [32] was identified in the index case. Validation and segregation of *C1QTNF5* p.Ser163Arg in the family revealed that, along with the five affected individuals, two of the family members initially considered unaffected also carried the mutation (S1 Fig, V:1 and IV:15). Table 3 summarizes causal mutations identified in the four families characterized.

**Table 3:** Mutations detected in the four families included in the study. adRP: autosomal dominant Retinitis Pigmentosa; AMD: age-related macular degeneration; adCRD: autosomal dominant cone-rod dystrophy; LORD: Late-onset retinal degeneration.

Family ID	Suspected diagnosis	Final diagnosis	Mutation	Reference
**RP-0107**	adRP	adCRD	*PRPH2 c556G>A; p*.*Asp186Asn (NM_000322*.*4)*	Kohl *et al*. 2012 [33]
**RP-0777**	adRP	adRP	*PRPF31 del 54*,*619*,*134 to 54*,*634*,*248 (NM_015629*.*3)*	This study
**RP-0858**	adRP + high myopia	xlRP	*RPGR c*.*485_486delTT; p*.Phe162Tyrfs*4 (NM_000328.*2)*	Sharon *et al*. 2000 [31]
**RP-0911**	adRP + AMD	LORD	*C1QTNF5 c*. *489C>A; p*.*Ser163Arg (NM_015645*.*3)*	Hayward C *et al*. 2003 [32]

### A novel large deletion spanning the entire *PRPF31* gene was identified in family RP-0777

**Fig 1:**
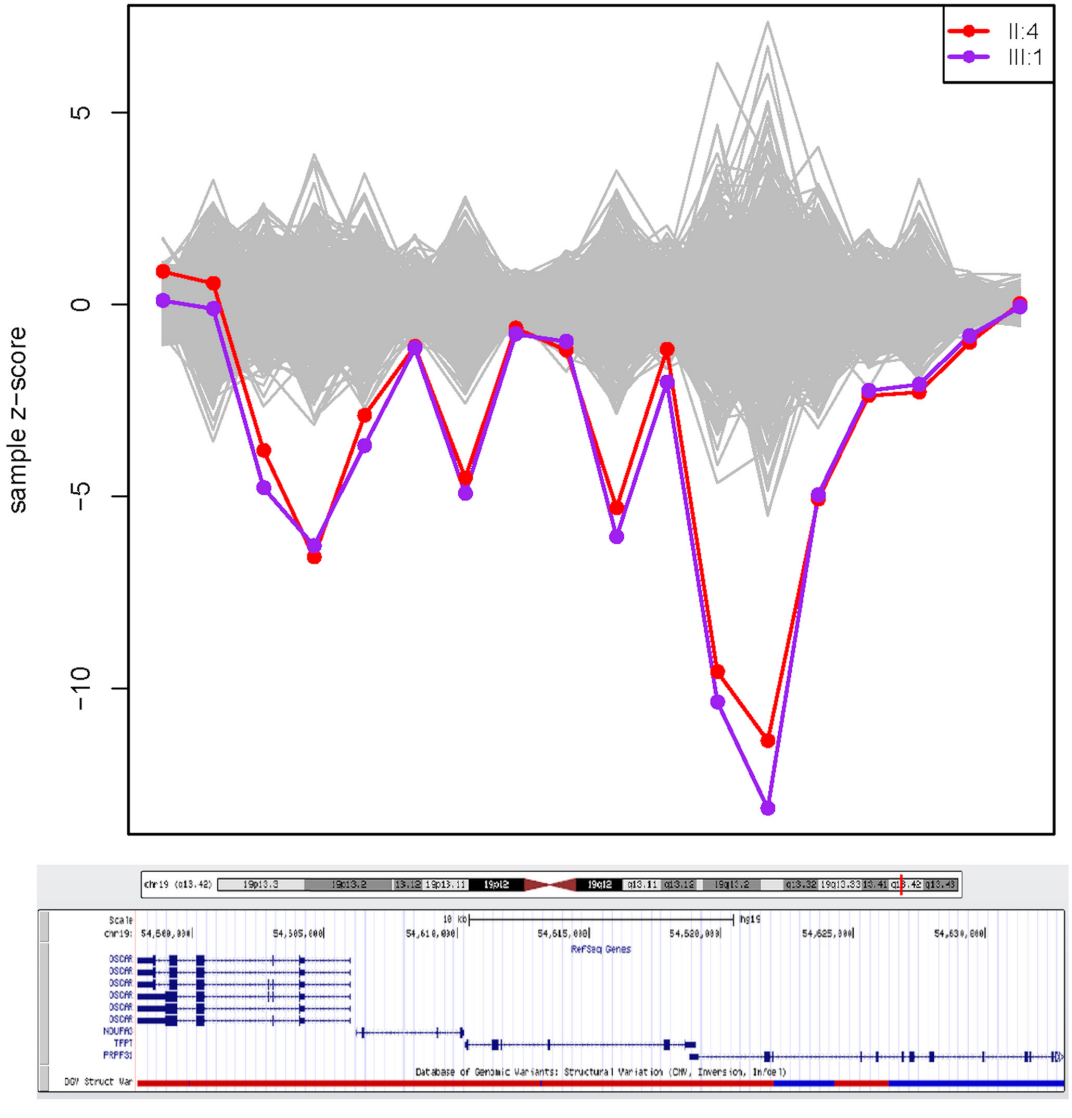
Region predicted by XHMM to harbor a heterozygous deletion. The x-axis represents the genome locus, with the genes and exons included in the algorithm, and the y-axis is the computed Z-score of PCA normalized read depth where positive values indicate predicted duplication and negative values deletion. The two affected individuals from family RP-0777 are highlighted with colors red (member II:4) and purple (member III:1) while grey lines represent the 663 control individuals used. Each point indicates a region containing an exon and they are paired with the corresponding exon/gene in a display of the region from the UCSC Genome Browser (http://genome.ucsc.edu/cgi-bin/hgGateway). On the left side of the UCSC Genome Browser track, the genes involved in the deletion are represented. From left to right, the genes/exons illustrated are exons 5 to 1 of the *OSCAR* gene, the entire genes *NUDFA3* and *TFPT* (4 and 6 exons, respectively), and exons 1 to 12 of *PRPF31*.

The presence of causative CNVs was investigated in the 3 families not characterized by SNP/*indel* analysis with the XHMM algorithm: RP-0502, RP-0777, and RP-1405 using 663 individuals from 212 families as controls. A heterozygous deletion in the region chr19: 54,600,186–54,628,017 (Fig 1) was identified in the two affected members of family RP-0777 with XHMM. The region identified by this prediction tool included the first exon of the gene *OSCAR*, the entire sequence of *NDUFA3* and *TFPT* and exons 1 to 7 of *PRPF31* (Fig 1).

For validation of the CNV predicted by XHMM in *PRPF31*, we used a TaqMan pre-designed probe in exon 1 and five UPL probes spanning the entire gene region: exon 1, and introns 3, 7, 8 and 13 (Table 2). Both CNV assays with pre-designed TaqMan and UPL probes confirmed that the coding sequence of *PRPF31* from chr19:54,618,875 to chr19:54,634,248 (exons 1 to 13) was hemizygously deleted in the two affected and present at two copies in the six unaffected members of the family. The phenotype of family RP-0777 is summarized in Table 2.

No CNV was identified in families RP-0502 and RP-1405.

### Screening of ORF15 in family RP-0502

In view of the results observed in family RP-0858 and due to the absence of male-to-male transmission in family RP-0502, we decided to screen this family for an X-linked inheritance model. Since *RPGR* was negative in this family (both by WES and gene-panels) and a large proportion of causal mutations of *RPGR* occur in the 3' end of the *ORF15* coding sequence, which was poorly covered in those sequencing assays, Sanger sequencing was used to scan for mutations in this gene. However, no mutation was found and therefore this family and family RP-1405, remain uncharacterized at the molecular level.

## Discussion

In the present study, we applied WES to six families with an initial diagnosis of adRP that had been extensively screened for known causative mutations and/or genes. Using this approach, we were able to characterize four out of the six families and, although the four families carried mutations in known genes, the identification of the genetic defect by WES led to the reappraisal of the phenotype from the initial adRP to xlRP in RP-0858, to LORD in RP-0911 and cone-rode dystrophy in RP-0107. This allowed the establishment of a correct diagnosis, estimation of risk recurrence and genetic counseling in these families. adRP was initially considered the most plausible phenotype based on the mode of inheritance in families, patients’ report on onset of symptoms such as night blindness, or visual acuity loss, and the ophthalmological data regarding fundus and visual field assessments. Clinical information was limited in some cases or exclusively recorded at later stages of the disease. This limitation, which is a common situation when studying this type of diseases, along with the clinical overlap of symptoms in different forms of inherited retinal dystrophies made establishing the precise diagnosis extremely complex and explains the reclassification of the phenotype in the families upon identification of the genetic cause. Therefore the use of a hypothesis free approach for mutation detection, such as WES, helps minimizing the impact that the availability of patient or family information has on the diagnostic success of retinal dystrophies.

Obligate carrier females of mutations in *RPGR* may display manifestations of the disease [8, 34], [35] and even be as severely affected as males [35, 36], as observed in family RP-0858, with all carrier females displaying a severe RP phenotype. What was remarkable in this family is that females affected largely outnumbered affected males (seven females versus three males). Additionally, the onset of symptoms was similar among both female and male carriers and there was no significant intra-familial variability in the symptomatology. These features are not often seen in xlRP and were along with the highly penetrant phenotype females presented the reasons why a dominant rather than an X-linked model was initially considered. Also, although myopia has been associated with xlRP caused by mutations in both *RPGR* and *RP2* [37–40], it is not exclusively found in this phenotype [41], and therefore was not used as a diagnostic criterion of xlRP. These results are consistent with the underestimated frequency of xlRP previously reported [35] and highlight the need of reviewing all adRP families with no male-to-male transmission, as already reported in [35], regardless of the severity of the symptoms, for testing of X-linked genes.

Family RP-0911 carried the mutation p.Ser163Arg *C1QTNF5* responsible for LORD, a very rare fully penetrant autosomal dominant retinal dystrophy with symptoms overlapping with a number of hereditary retinal conditions [42]. The phenotype of LORD evolves with time and in early stages can be misdiagnosed as early age-related macular degeneration (AMD), and later on with RP [42, 43], [44],[45], [46]. Due to the limited clinical data available in this family and the lack of literature about this entity by the time this family was first evaluated, LORD was never considered and different diagnoses were attributed to the three affected members from RP-0911: RP in IV:1, age-related macular degeneration in IV:6, and both in IV:11, initially suspecting of two distinct entities co-segregating in the family. Further clinical re-evaluation of the family evidenced that some of the symptoms were consistent with what has been reported in families affected with LORD whereas others, like neovascularization, was not present in any member of the family at the time of diagnosis. Interestingly, member IV:10, who had referred night blindness as the only symptom when she was 59 years, had evidence of drusen in the eye fundus at time of testing (62 years), but the rest of the ophthalmological study was completely normal. Unfortunately, we do not have information on the progression of the phenotype in this family, so a detailed description of the symptomatology and progression of the disease cannot be provided in this study.

WES also helped in the correct diagnosis of family RP-0107, who was found to harbor a mutation in *PRPH2* previously identified causing autosomal dominant cone dystrophy [30]. All affected members from family RP-0107 presented eye fundus and visual fields compatible with RP plus macular affectation, except for individual V:4 that had a diffuse retinal degeneration. Because the family was studied in the latest stages of the disease, the phenotype at that time was more compatible with RP than cone-rode dystrophy. Remarkably, in our diagnosis algorithm of adRP, *PRPH2* is one of the first genes to be screened, however in the index case of this family this was done by DGGE and possibly due to the sensitivity of the methodology, the mutation was not detected.

A large deletion spanning the entire region of *PRPF31* was detected in family RP-0777 using exome data. *PRPF31* is one of the most frequently mutated genes in adRP, accounting for 5–10% of the cases and with large deletions being responsible for almost 3% of the cases [34]. This deletion found in this study is similar to that reported by Kohn et al. [47] that besides *PRPF31* also included the genes *OSCAR*, *NUDFA3*, and *TFPT*. However, they found the breaking point of the CNV in intron 11 and in our family, the deletion expands up to intron 13, at least, as evidenced by experimental validation with the UPL assays. The phenotype found in RP-0777, summarized in Table 3, is very similar to that reported by Kohn and colleagues [47] and surprisingly mild given the size of *PRPF31* deleted. Interestingly, despite NGS being widely used in search for mutations in retinal dystrophies, so far only Eisenberger *et al*. [48] and Nishiguchi *et al*. [49] have reported the discovery of large deletions in genes causing retinal dystrophies using sequencing data. With this study we support the feasibility of detecting CNV in genes responsible for retinal dystrophy using NGS, thus expanding the potential of this tool in the diagnosis of these diseases.

To our knowledge, WES has not been applied systematically to date as diagnostic tool in dominant forms of retinal dystrophies. This study was part of a larger multi-center sequencing effort on Mendelian disorders where WES has been successful identifying the genetic cause of a number of phenotypes, including the discovery of a new genetic cause of a syndromic form of RP in one of our Spanish families [50]. However, the results from the current study, with only known genes identified, indicate that WES may be an adequate and efficient tool once all known genetic causes of retinal dystrophy have been ruled out. For that purpose, targeted sequencing is regarded now as the most optimal approach of candidate gene screening [7, 10, 15, 51]. Almost simultaneous to this project, our group developed an NGS custom panel with 73 genes related to retinal dystrophies that was applied to 59 index cases of families with adRP, including the non-characterized families RP-0502 and RP-1405 [10]. The authors found a detection rate of 27% with 64% of the cases carrying new mutations in known genes, which is in line with previous results of studies performed in similar conditions [7], [8]. Very recently, Consugar et al. published a comparative analysis on the performance of panel-based versus WES [15]. The authors concluded that targeted sequencing was more sensitive for variant detection than WES, with a superior and more even coverage of genes, and therefore the preferred method for genetic diagnostic testing [15].

Based on our experience over the past years on the use of NGS technologies and in line with previous reports [7, 15, 51], we propose to start the diagnostic testing with targeted sequencing of candidate genes, due to the methodological and cost advantages over WES. As a second step, due to the high incidence of xlRP among families initially classified as dominant ([35] and this study), we propose to screen *ORF15* in any family showing no male-to-male transmission, regardless of the symptomatology or the number of females affected, to rule out a possible X-linked inheritance. *ORF15* is responsible for up to 60% of xlRP disease-causing mutations [52], and because of its high repetitive nature is not usually adequately covered by NGS methods. Finally, once known causes of inherited retinal dystrophies have been ruled out either WES or WGS may be used in the almost 50% of remaining cases that are estimated to harbor mutations in rarer novel genes [3].

## Supporting Information

S1 Fig Pedigrees of the six families studied. + and +/+: wild type genotypes. m: mutation detected in hemizygosis; m/+: mutation detected in heterozygosis. Filled and unfilled symbols represent affected and unaffected individuals respectively. Squares indicate males and circles females. Arrows indicate the index cases. Red circles represent individuals subjected to whole exome sequencing.
(TIFF)

S2 Fig Eye fundus images from members of families RP-0777 and RP-0911. Each patient presents two fundus images per eye. A) Family RP-0777 and B) Family RP-0911. RE = Right eye. LE = Left eye.
(TIFF)

S3 Fig Electroretinogram recordings from members of families RP-0777 and RP-0911. (TIFF)

S1 Table Genetic screening performed to the six families prior to whole exome sequencing. **SSCP:** Single Strand Conformation Polymorphism; **DGGE:** CG-clamped Denaturing Gradient Gel Electrophoresis. The parentheses indicate the exons targeted by these techniques; otherwise the entire gene was screened. For ADRP Chip**, v**ersion 1 includes 355 SNPs in *CA4*, *CRX*, *FSCN2*, *IMPDH1*, *NR2E3*, *NRL*, *PRPF3*, *PRPF31*, *PRPF8*, *PRPH2*, *RHO*, *ROM1*, *RP1*, *RP9*, *TOPORS*; and *v*ersion 2 includes 414 SNPs in *CA4, CRX, FSCN2, IMPDH1, KLHL7, NR2E3, NRL, PRPF3, PRPF31, PRPF8, PRPH2, RHO, ROM1, RP1, RP9, TOPORS; Sanger sequencing was used to screen mutations in exons 16 and 25 for SNRNP200, exon 2 for NR2E3 and exon 13 for GUCY2D. For IMPDH1 all exons were sequenced. RD_NGS_Panel refers to the custom Next Generation Sequencing panel from* S2 Table.
(DOCX)

S2 Table Genes associated with RP and LCA included in the customized RD_NGS_Panel. Macular dystrophy (MD); retinitis pigmentosa (RP); Leber's congenital amaurosis (LCA); congenital stationary night blindness (CSNB); choroideremia (CHM), cone-rod dystrophy (CORD); autosomal recessive (ar); autosomal dominant (ad); X-linked (xl); McKusick-Kaufman syndrome (MKKS); Senior Loken Syndrome (SLS); vitreoretinopathy proliferative (VRP); enhanced S-cone syndrome (ESC).
(DOCX)

S3 Table Primers and conditions used for PCR and sequencing of *ORF15*. (DOCX)
