# Supplementary figures and images for: Application of Whole Exome Sequencing in Six Families with an Initial Diagnosis of Autosomal Dominant Retinitis Pigmentosa: Lessons Learned
Source: PLoS One. 2015 Jul 21;10(7):e0133624. doi: 10.1371/journal.pone.0133624 (PMC4509755; doi:10.1371/journal.pone.0133624)

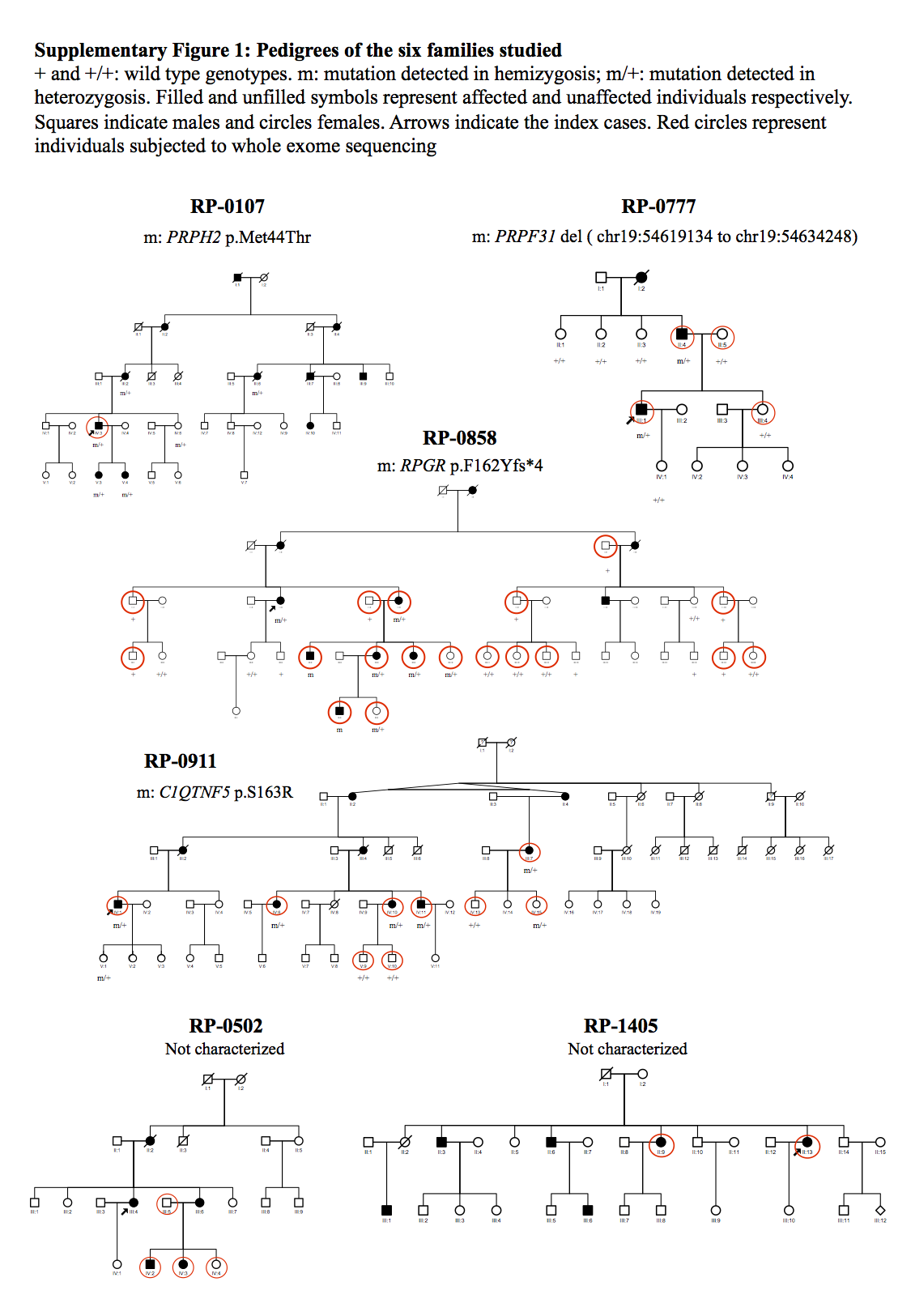

Supplement: S1 Fig — + and +/+: wild type genotypes. m: mutation detected in hemizygosis; m/+: mutation detected in heterozygosis. Filled and unfilled symbols represent affected and unaffected individuals respectively. Squares indicate males and circles females. Arrows indicate the index cases. Red circles represent individuals subjected to whole exome sequencing. (TIFF) [file pone.0133624.s001.tiff]

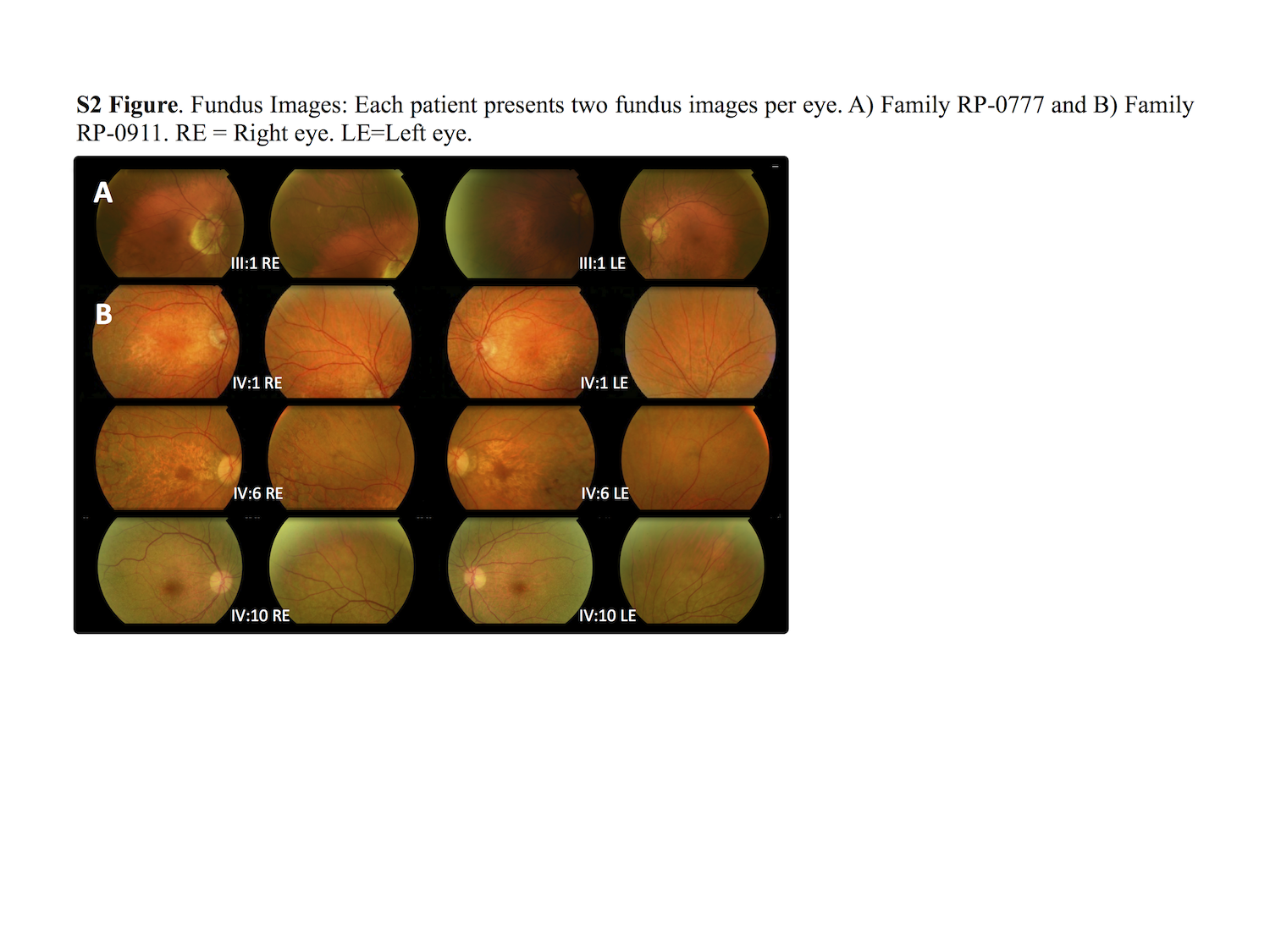

Supplement: S2 Fig — Each patient presents two fundus images per eye. A) Family RP-0777 and B) Family RP-0911. RE = Right eye. LE = Left eye. (TIFF) [file pone.0133624.s002.tiff]

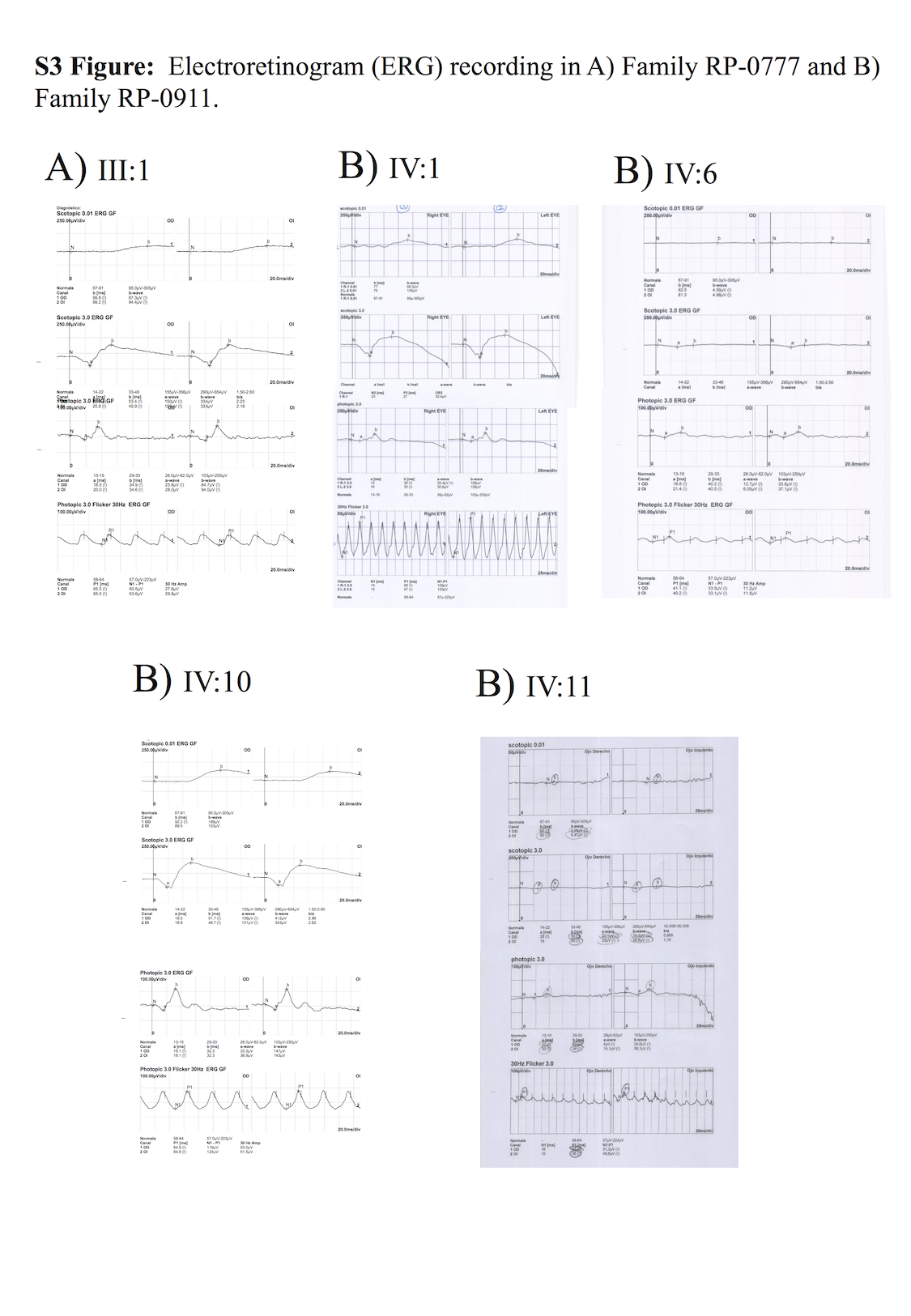

Supplement: S3 Fig — (TIFF) [file pone.0133624.s003.tiff]
