# Supplementary material for: Application of Whole Exome Sequencing in Six Families with an Initial Diagnosis of Autosomal Dominant Retinitis Pigmentosa: Lessons Learned
Source: PLoS One. 2015 Jul 21;10(7):e0133624. doi: 10.1371/journal.pone.0133624 (PMC4509755; doi:10.1371/journal.pone.0133624)
Supplement: S1 Table — SSCP: Single Strand Conformation Polymorphism; DGGE: CG-clamped Denaturing Gradient Gel Electrophoresis. The parentheses indicate the exons targeted by these techniques; otherwise the entire gene was screened. For ADRP Chip, version 1 includes 355 SNPs in CA4, CRX, FSCN2, IMPDH1, NR2E3, NRL, PRPF3, PRPF31, PRPF8, PRPH2, RHO, ROM1, RP1, RP9, TOPORS; and version 2 includes 414 SNPs in CA4, CRX, FSCN2, IMPDH1, KLHL7, NR2E3, NRL, PRPF3, PRPF31, PRPF8, PRPH2, RHO, ROM1, RP1, RP9, TOPORS; Sanger sequencing was used to screen mutations in exons 16 and 25 for SNRNP200, exon 2 for NR2E3 and exon 13 for GUCY2D. For IMPDH1 all exons were sequenced. RD_NGS_Panel refers to the custom Next Generation Sequencing panel from S2 Table. (DOCX) [file pone.0133624.s004.docx]

**S1 Table. Genetic screening performed to the six families prior to whole exome sequencing.**

Legend: **SSCP:** Single Strand Conformation Polymorphism; **DGGE:** CG-clamped Denaturing Gradient Gel Electrophoresis. The parentheses indicate the exons targeted by these techniques; otherwise the entire gene was screened. For ADRP Chip**®, v**ersion 1 includes 355 SNPs in *CA4, CRX, FSCN2, IMPDH1, NR2E3, NRL, PRPF3, PRPF31, PRPF8, PRPH2, RHO, ROM1, RP1, RP9, TOPORS;* and *v*ersion 2 includes 414 SNPs in *CA4, CRX, FSCN2, IMPDH1, KLHL7, NR2E3, NRL, PRPF3, PRPF31, PRPF8, PRPH2, RHO, ROM1, RP1, RP9, TOPORS; Sanger sequencing was used to screen mutations in exons 16 and 25 for SNRNP200, exon 2 for NR2E3 and exon 13 for GUCY2D. For IMPDH1 all exons were sequenced. RD_NGS_Panel refers to the custom Next Generation Sequencing panel from S2 Table.*

|  | **SSCP/DGGE** | **ADRP Chip® version** | **Genes subjected to Sanger sequencing** | **RD_NGS_Panel** |
| --- | --- | --- | --- | --- |
| **RP-0107** | *CA4 (3, 4, 6), CRX, ELOVL4, FSCN2, NRL PRPF3, PRPF8 (42), PRPF31 (8), PRPH2, RHO, ROM-1* | Version 2 | *IMPDH1, SNRNP200* | No |
| **RP-0502** | *CRX, ELOVL4, FSCN2, IMPDH1, PRPH2, RHO, ROM-1, NRL, PRPF8 (42), PRPF31 (8)* | Version 1 | *IMPDH1, NR2E3*, *PRPH2, SNRNP200* | Yes |
| **RP-0777** | *CA4 (3, 4, 6) CRX, PRPH2, RHO, PRPF8 (42), PRPF31 (8)* | Version 2 | *IMPDH1, NR2E3*, *PRPH2, SNRNP200* | No |
| **RP-0858** | *CA4 (3, 4, 6), CRX, PRPH2, RHO, PRPF8 (42), PRPF31 (8)* | Version 2 | *IMPDH1, NR2E3*, *PRPH2, SNRNP200* | No |
| **RP-0911** | *CA4 (3, 4, 6), CRX, PRPH2, RHO, PRPF8 (42), PRPF31 (8)* | Version 1 | *GUCY2D, IMPDH1*, *NR2E3*, *SNRNP200* | No |
| **RP-1405** | NA | Version 2 | *GUCY2D, IMPDH1*, *NR2E3*, *SNRNP200* | Yes |
