# Supplementary material for: Application of Whole Exome Sequencing in Six Families with an Initial Diagnosis of Autosomal Dominant Retinitis Pigmentosa: Lessons Learned
Source: PLoS One. 2015 Jul 21;10(7):e0133624. doi: 10.1371/journal.pone.0133624 (PMC4509755; doi:10.1371/journal.pone.0133624)
Supplement: S2 Table — Macular dystrophy (MD); retinitis pigmentosa (RP); Leber's congenital amaurosis (LCA); congenital stationary night blindness (CSNB); choroideremia (CHM), cone-rod dystrophy (CORD); autosomal recessive (ar); autosomal dominant (ad); X-linked (xl); McKusick-Kaufman syndrome (MKKS); Senior Loken Syndrome (SLS); vitreoretinopathy proliferative (VRP); enhanced S-cone syndrome (ESC). (DOCX) [file pone.0133624.s005.docx]

**S2 Table:** Genes associated with RP and LCA included in the customized

Legend: RD_NGS_Panel. Macular dystrophy (MD); retinitis pigmentosa (RP); Leber's congenital amaurosis (LCA); congenital stationary night blindness (CSNB); choroideremia (CHM), cone-rod dystrophy (CORD); autosomal recessive (ar); autosomal dominant (ad); X-linked (xl); McKusick-Kaufman syndrome (MKKS); Senior Loken Syndrome (SLS); vitreoretinopathy proliferative (VRP); enhanced S-cone syndrome (ESC).

| **Gene** | **cDNA sequence** | **Number of targets** | **% Coverage of design** | **Disease** | **Inheritance** | **Other described phenotype** |
| --- | --- | --- | --- | --- | --- | --- |
| *ABCA4* | NM_000350.2 | 50 | 100% | MD | ar | arRP/arCORD |
| *ABHD12* | NM_001042472.2 | 14 | 100% | RP | ar | RP+ataxia+hearing loss |
| *ADAMTS18* | NM_199355.2 | 23 | 99.6% | RP | ar |  |
| *AIPL1* | NM_014336.3 | 6 | 95.6% | LCA | ar | adCORD |
| *BBS1* | NM_024649.4 | 17 | 100% | ciliopatia | ar | Bardet-Biedl / arRP |
| *BEST1* | NM_004183.3 | 11 | 100% | MD | ad / ar | arRP / adRP / ad vitreoretinochoroidopathy/ ar bestrophinopathy |
| *C2orf71* | NM_001029883.2 | 2 | 100% | RP | ar |  |
| *C8ORF37* | NM_177965.3 | 6 | 100% | RP | ar | CORD |
| *CA4* | NM_000717.3 | 8 | 97.0% | RP | ad |  |
| *CABP4* | NM_145200.3 | 6 | 98.3% | CSNB | ar | arCORD / LCA |
| *CEP290* | NM_025114.3 | 55 | 99.4% | LCA | ar | SLS/ Joubert / MKKS |
| *CERKL* | NM_001030311.2 | 14 | 98.0% | RP | ar | arCORD with inner retinopathy |
| *CHM* | NM_000390.2 | 16 | 99.0% | CHM | xl |  |
| *CLRN1* | NM_174878.2 | 5 | 100% | Usher | ar | arRP |
| *CNGA1* | NM_001142564.1 | 12 | 99.9% | RP | ar |  |
| *CNGB1* | NM_001297.4 | 34 | 99.9% | RP | ar |  |
| *CRB1* | NM_201253.2 | 12 | 100% | LCA | ar | arRP |
| *CRX* | NM_000554.4 | 4 | 95.5% | LCA | ar / ad | adRP / adCORD |
| *CYP4V2* | NM_207352.3 | 11 | 97.6% | RP | ar | Bietti crystalline corneoretinal dystrophy |
| *DHDDS* | NM_024887.3 | 9 | 100% | RP | ar |  |
| *EYS* | NM_001142800.1 | 45 | 99.2% | RP | ar |  |
| *FAM161A* | NM_032180.2 | 7 | 100% | RP | ar |  |
| *FSCN2* | NM_001077182.2 | 5 | 100% | RP | ad |  |
| *GUCA1B* | NM_000180.3 | 4 | 96.2% | RP | ad |  |
| *GUCY2D* | NM_000180.3 | 20 | 98.5% | LCA | ar | adRP / adCORD |
| *IDH3B* | NM_006899.3 | 12 | 100% | RP | ar |  |
| *IMPDH1* | NM_000883.3 | 17 | 100% | RP | ad |  |
| *IMPG1* | NM_001563.2 | 17 | 100% | RP | ar |  |
| *IMPG2* | NM_016247.3 | 19 | 99.7% | RP | ar |  |
| *IQCB1* | NM_001023570.2 | 15 | 99.8% | LCA | ar | SLS |
| *KCNJ13* | NM_002242.4 | 3 | 100% | LCA | ar | adVRP |
| *KLHL7* | NM_001031710.2 | 14 | 100% | RP | ad |  |
| *LCA5* | NM_181714.3 | 9 | 99.9% | LCA | ar |  |
| *LRAT* | NM_004744.3 | 3 | 97.8% | RP | ar | LCA |
| *MAK* | NM_001242957.1 | 13 | 98.2% | RP | ar |  |
| *MERTK* | NM_006343.2 | 19 | 100% | RP | ar | LCA / arCORD |
| *MPDZ* | NM_003829.4 | 45 | 100% | RP | ar |  |
| *NMNAT1* | NM_022787.3 | 5 | 93.4% | LCA | ar |  |
| *NR2E3* | NM_014249.2 | 8 | 100% | RP | ar / ad | arESC; Goldmann-Favre syndrome; combined adt and ar retinopathy |
| *NRL* | NM_006177.3 | 3 | 100% | RP | ar / ad |  |
| *OFD1* | NM_003611.2 | 24 | 100% | XLRP | xl | Joubert |
| *PDE6A* | NM_000440.2 | 22 | 98.4% | RP | ar |  |
| *PDE6B* | NM_000283.3 | 23 | 99.5% | RP | ar | ad CNSB |
| *PDE6G* | NM_002602.3 | 4 | 100% | RP | ar |  |
| *PRCD* | NM_001077620.2 | 6 | 100% | CORD | ar | arRP |
| *PROM1* | NM_006017.2 | 29 | 100% | MD | ad / ar | arRP/ adCORD |
| *PRPF3* | NM_004698.2 | 16 | 100% | RP | ad |  |
| *PRPF31* | NM_015629.3 | 15 | 97.4% | RP | ad |  |
| *PRPF6* | NM_012469.3 | 21 | 100% | RP | ad |  |
| *PRPF8* | NM_006445.3 | 43 | 99.8% | RP | ad |  |
| *PRPH2* | NM_000322.4 | 3 | 100% | RP | ad | adMD / adCORD / digenic retinitis pigmentosa with ROM1 |
| *RBP3* | NM_002900.2 | 4 | 100% | RP | ar |  |
| *RD3* | NM_183059.2 | 3 | 100% | LCA | ar |  |
| *RDH12* | NM_152443.2 | 9 | 100% | LCA | ar / ad | arRP/adRP |
| *RGR* | NM_001012720.1 | 7 | 91.0% | RP | ar | ad choroidal sclerosis |
| *RHO* | NM_000539.3 | 5 | 100% | RP | ad / ar | ad CNSB |
| *RLBP1* | NM_000326.4 | 9 | 100% | RP | ar | arCORD / ar Bothnia dystrophy / ar retinitis punctata albescens |
| *ROM1* | NM_000327.3 | 3 | 100% | RP | ad | digenic retinitis pigmentosa with PRPH2 |
| *RP1* | NM_006269.1 | 4 | 100% | RP | ad / ar |  |
| *RP2* | NM_006915.2 | 5 | 98.4% | RP | xl |  |
| *RP9* | NM_203288.1 | 6 | 84.6% | RP | ad |  |
| *RPE65* | NM_000329.2 | 14 | 98.0% | LCA | ar | arRP |
| *RPGR* | NM_001034853.1 | 15 | 84.8% | RP | xl | xlCORD / xlMD |
| *RPGRIP1* | NM_020366.3 | 24 | 100% | LCA | ar | arCORD |
| *SAG* | NM_000541.4 | 16 | 99.6% | RP | ar | CNSB / ar Oguchi disease |
| *SEMA4A* | NM_022367.3 | 16 | 100% | RP | ad | adCORD |
| *SNRNP200* | NM_014014.4 | 45 | 99.6% | RP | ad |  |
| *SPATA7* | NM_018418.4 | 12 | 98.9% | LCA | ar | arRP |
| *TOPORS* | NM_005802.4 | 3 | 100% | RP | ad |  |
| *TTC8* | NM_144596.2 | 15 | 100% | RP | ar | Bardet-Biedl syndrome |
| *TULP1* | NM_003322.3 | 15 | 100% | RP | ar | LCA |
| *USH2A* | NM_206933.2 | 73 | 100% | RP | ar | Usher |
| *ZNF513* | NM_144631.5 | 4 | 100% | RP | ar |  |
