# Supplementary material for: Application of Whole Exome Sequencing in Six Families with an Initial Diagnosis of Autosomal Dominant Retinitis Pigmentosa: Lessons Learned
Source: PLoS One. 2015 Jul 21;10(7):e0133624. doi: 10.1371/journal.pone.0133624 (PMC4509755; doi:10.1371/journal.pone.0133624)
Supplement: S3 Table — (DOCX) [file pone.0133624.s006.docx]

| **Amplicon** | **Forward Primer (5’-3’)** | **Reverse Primer (5’-3’)** | **Product Size (bp)** | **PCR Annealing Temperature (ºC)** |
| --- | --- | --- | --- | --- |
| 1 | GTAGATAAGTTGTCCTTGTC | TTCGATAGTCGTAGCTGGC | 226 | 54 |
| 2 | AATGAAAGAAGGGAAAGCATG | TGCTTCTGATTCCTTCTGAC | 258 | 55 |
| 3 | GGTGTTGATCAACTTGATG | CACATCTTGCTTGCAACTTTC | 264 | 54 |
| 4 | GAGGTATGAATACTGAGAGTG | ATTTCCTGCCATACCGTATG | 249 | 55 |
| 5 | ATGGATTCCAGCAGCCTGAG | CCTTTTGAATCCTCTGCTCC | 203 | 58 |
| 6 | GATCGCTTGTCAGAGATCCC | ACTACCTTCCTCACAGTTC | 249 | 56 |
| 7 | AGAAGCAGAGGATGGGCCTG | CTTTCCTTCTGATGGCCCTG | 234 | 62 |
| 8 | GAATGGAAGAAGAGGGATGG | TTTTCACGTTCTCCCTCCAC | 219 | 56 |
| 9 | GGAGGAGGAGCATGGAGAAG | CCTCTTCCCCCTCTCCTTGG | 203 | 58 |
| 10 | GTGGAGGGAGAACGTGAAAAG | CCCTCCCCTTCCTCCTCTTC | 236 | 67 |
| 11 | AGGAGAGGAAGAAGGAGACC | TTCTTCGCCTGTCTCCTGATA | 868 | 63 |
| 11_int * | ---- | TTCCCCCTCCCCTTCTCCA |  |  |
| 12 | GATCTGTGAAATATGGCAAAC | GACTGGCCATAATCGGGTCAC | 215 | 54 |
| 13 | GGAGGAAGAAGAGGGGAAGTATCA | CCCTGTGTGTTAGTAACTGAC | 154 | 58 |

**S3 Table. Primers and conditions used for PCR and sequencing of *ORF15***

* An internal primer for sequencing of amplicon 11 was also used.
